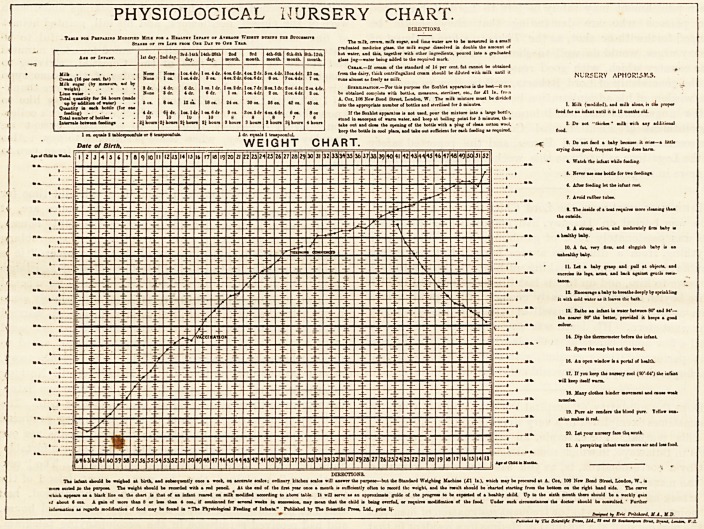# "The Hospital" Nursing Mirror

**Published:** 1900-08-18

**Authors:** 


					The HospitalAugust 18, 1900.
" ?lie 3l?osj>ttnl" iltivstng trior*
Being tiie Nursing Section of "The Hospital."
{Contributions for this Section of "The Hospital" should be addressed to the Editor, The Hospital, 28 & 29, Southampton Street, Strand,
London, W.O., and should have the word " Nursing:" plainly written in left-hand top corner of the envelope.]
Iflotes on IRews from tbe Tfturslng Worlfc.
TWENTY-EIGHT NEW NURSES FOR SOUTH
AFRICA.
"We are informed by tlie Secx*etary of State for War
that a further detachment of members of the Army
Nursing Service Reserve will leave for South Africa
?about the 20th inst., consisting of Nursing Sisters G.
Black, E. M. Beetham, R. M. Browse, E. L. Cash,
L. A. Clarke, A. R. Chitty, M. Everett, M. Forrest, E.
?Gray, A. M. Guttridge, A. E. Holmes, A. E. Howard,
M. J. Hutcheson, D. B. Hyland, A. Jones, M. John-
stone, A. Matheson, A. Mackenzie, E. M. Mazzuchi, C.
Meany, M. C. Meeke, E. S. Owen, M. Parsons,
I". E. Ridgley, M. E. Richardson, J. H. Roberts,
E. M. Sutton, and A. G. Wason. Miss Lucy
Alice Clarke was trained at the Royal Free Hos-
pital for four years, and has been Charge
Nurse at Park Hospital, Hither Green, since August,
1899. Miss A. R. Chitty has been Matron at Stroud
Hospital, but has lately been on the staff at Netley.
Miss Catherine Meany was trained at the Royal
Hospital, Salford, where she has been Sister since 1888.
Miss Elizabeth Susannah Owen was trained at the
Royal Infirmary, Liverpool. She was engaged in
private nursing from September, 1885, to April, 1891.
She has since been Staff Nurse at Toxtetlx Park Infir-
mary, Liverpool; Union Infirmary, Sheffield; Gore
Farm Hospital, Darenth ; Superintendent Nurse at the
Union Infirmary, Luton ; and Superintendent Nurse at
the Stepney Union Infirmary, Bromley-by-Bow. Miss
Owen holds the L.O.S. certificate.
THE NEW SOMERSET HOSPITAL AT CAPE
TOWN.
On Wednesday, June 20tli, an interesting ceremony
took place in connection with the Somerset Hospital,
?Cape Town, when the new Victoria wing was opened
by the Mayor. This hospital occupies an imposing site
facing the bay, and is the first object which attracts
one's attention on nearing Cape Town. The foundation-
stone of the main building was laid by Sir George Grey
*0. 1859; it remained a Government hospital for some
years, but was subsequently handed over to the public;
^nd now, with the addition of the new wing, and an
adjoining building known as " The Bungalow," pro-
Vldes accommodation for both white and coloured
patients. Some paying patients are received, but the
majority of the beds are free, and each bed costs the
mstitution the sum of 4s. 9d. per diem. The nursing
Arrangements are under the supervision of a Sister
Superior of the All Saints' Community. At the open-
1Qg function the nux'ses, looking bright and fresh in
their blue linen dresses, gathered in the verandah round
tbe Sister Superior, many of them wearing button-holes
?f flowers or knots of red, white, and blue x-ibbon in
honour of the occasion. In the " Lady Loch" ward
^ea was dispensed to the visitors by the nurses and
other ladies. This px*etty ward was beautifully deco-
rated with the flowers for which South Africa is famed.
The walls are panelled with paintings in oil colours,
contributed by some of the many friends of the insti-
tution, and which are calculated to give much pleasure
to the small coffee-coloured occupants of the dainty
cots. The hospital has undergone considerable strain
during the last few months, and the managers, in
thanking the public for their gifts of fruit, egga, &c.,
which, owing to the exigencies of the war, are very
scarce in Cape Town, expressed a hope that the support
which has been given to meet the special need of the
hospital in time of war might be continued when peace
was restored to South Africa.
NURSING CONVALESCENTS ON THE
"ENGLISHMAN."
The Sister in-Charge of the "Englishman," which
has recently arrived, after a long passage of 34 days,
says that the weather was beautiful until the last few
days. There were 650 men on board, nearly all con-
valescent fever cases. The " Englishman " is a horse
boat, but everything was very clean, and splendidly
arranged. The wards (they were really more like
barracks, for the men were well enough to be up) were
on the starboard side of the quarter-deck. With Mrs.
Bourchier were two " Africander," or British Colonial,
Sisters, and, like the staff of the " Avonmore Castle,"
they had very little work to do. One of the Sisters
took night duty, and a ward-master and three orderlies
were in each ward. Sister Bourchier has been through
more than one campaign, and no doubt her Egyptian
experiences stood her in good stead in South Africa.
For two months she was continually under fire, as she
was in charge of the Convent in Ladysmith, for which
she volunteered, though she had retired from active
service in 1886.
"Many of the officers and men," she tells us, "cannot
now hear the whistle for a hansom without a shock, for it
was the signal given by native boys whenever the smoke of
Long Tom was seen, and then everyone made for cover as
quickly as possible."
At this convent Mrs. Bourchier had everything to do
herself, even to gathering the wood for lighting her
fires to cook by; everyone was so hard at work that
she had not even a native boy to fetch and carry, Yet
she only lost one of her patients. When the relief
column came she had not tasted food for forty-eight
hours. She couldn't touch horse-flesh, though she was
obliged to cook it for the men; there was nothing else.
She was nursing with a temperature of 103. The
convent was a civilian hospital, but army rations were
supplied. On the voyage home Mrs. Bourchier invented
various ways of amusing the men by making cushions,
wall bags, &c., which were raffled, the proceeds going
for tobacco.
THE VOYAGE OF THE "GREEK."
The ss. " Greek," which arrived from Durban on the
7th, had nearly .300 men on board belonging to various
branches of the service. Typhoid, dysentery, and a few
264
" THE HOSPITAL " NURSING MIRROR.
The Hospital,
Aug. 18, 1900.
cases of wounds were the chief complaints, but as all
were convalescent the work of the two nursing sisters
was very light. For the most part the men steadily
improved in health. " They invariably made the best of
it," says Sister Waller, "and laughed over the many
discomforts incidental to a third class passage home, not
uttering a word of complaint even when the ship did
roll." Sister Waller is warm in her praise of both officers
and men. " Captain Armstrong and his officers were
extremely kind to us all, and I spent a most happy
time on board the' Greek.' We had sports and concerts,
each man doing his best for the pleasure and comfort of
the others. Sister Waller was for three years on the staff
of the Kimberley Hospital. She left it on February 18tb,
and was in camp from that time until the " Greek " left
Durban in July. She speaks most highly of the order
and good management preserved throughout the siege.
The hospital, supported mainly by the De Beers Mining
Company, was the only one in the town for white
patients ; 170 out of 270 beds were set aside for wounded
and sick of the regular forces. Sister Waller testifies to
the patience of the men in hospital, and mentions that
she has come across numberless cases of men nursing
each other almost as well as a woman could have done,
" with only their kind hearts and willing hands to tell
them what to do.''
WOMEN NURSES NEAR THE FRONT.
An Army Sister writes from Maritzburg to urge
" that in future white wars both hospitals and women
nurses should be carried nearer the front." She says :
" Of course, we realise that proper accommodation for
women nurses could not be provided within the fighting
area; but it is better that the temporary comfort of a
few dozen strong, healthy young women should be
sacrificed than that hundreds of suffering sick soldiers
should be neglected, killed outright, or permanently
injured for want of proper care. Doubtless it would
happen with a feminine nursing staff near the front
that after a defeat they would be taken prisoners with
their patients. In case of retreat they would have to
be left behind with the sick. What matter? It is
doubtful whether the enemy would want to hamper
themselves with feminine impedimenta. Anyway, the
women would not be murdered or ill-treated?in a
white man's land, at any rate?any more than the sick
and wounded are."
"HARD ON US."
Everyone has heard about the " silly women " who
brought down upon their heads the wrath of Mr. Treves
and others by going out to South Africa without hos-
pital training, and without experience, and who only
succeeded in being in everybody's way. But there are
others, with much experience and some amount of hos-
pital training, who have been employed as nurses,
and it is about these that the words at the head of this
paragraph were spoken by a nursing sister just returned
from the Cape. She did not in the least deprecate their
work; she simply said, " It's hard on us."
TURKS AND TRAINED NURSES.
The interesting fact is reported from New York that
four trained nurses have been in attendance at the
Turkish Legation in the city. The origin was an
attack of typhoid fever, to which the Turkish Minister,
Ali Ferrouh Bey, fell a victim. An American doctor
was summoned, and he engaged two female nurses to
attend the patient. Then, the Ambassador's brother-
in-law broke his leg, and another trained nurse was-
called in. Lastly, -while the three nurses were at the
Embassy, Madame Ferrouh presented her husband with
a son, and the services of a fourth were secured.
A SCANDAL AT SHEPTON MALLET.
A shameful state of affairs prevails, or has pre-
vailed, at Shepton Mallet Infirmary. We learn from a
report of a meeting of the Board of Guardians in the
Bristol Times that Nurse Dobson, a newly appointed
nurse, came before the Board and made very serious
allegations as to the cleanliness of the infirmary wards.
She said " the middle of the wards were clean, and that
was all." ..." Under the beds were filthy linen,
pieces of mouldy suet, and other things, and some of the
inmates, she alleged, had not had a change of under-
clothing for a month. She had cleaned some of the foul
places herself, because she was afraid of catching
typhoid if she had not cleansed these places. If
she had known the state the house was in she would
not have taken the post for ?50 a year." This is very out-
spoken language, and the matron, who was interrogated
on the matter, made a strange answer. She said " that
the previous nurse was very dirty, and allowed the wards
to get into their present dirty condition, though they
were not so bad as described by Nurse Dobson." But
the matron herself admitted that they were dirty. Why,
then, did she not take steps to make them clean, instead
of leaving it to a new nurse to undertake the duty p The
state of things which Miss Dobson depicts supplies yet
another reason for the indisposition of good nurses to
place their services at the disposal of boards of
guardians.
SAVED BY A NURSE.
The North American contains the following illustra-
tion of the resourcefulness and persistence of a nuree:
" Miss Rebecca Robinson, who recently graduated from
the Nurses' Training School of the Jewish Hospital,
Philadelphia, is the heroine of an interrupted tragedy
at Atlantic City. Taking an early morning stroll along
the beach, Miss Robinson met near Mississippi Avenue
a party of men carrying between them the limp body
of another man. The young nurse hastened toward the-
group, and asked what had happened. ' The man has
just been drowned,' replied one of the group, ' and we
are taking him to the hotel at which he was staying.'
' Let me see him,' she said. The carriers were disinclined
to accede to her request, but after persistent appeals
their burden was laid at the feet of the nurse, who at
once began scientific methods of artificial respiration*
Then hot whisky and spirits of ammonia were secured at
her direction and applied to the blue lips of the patient.
After a half-hour of vigorous work the supposed corpse
suddenly opened his eyes. In another half-hour he was
able to get upon his feet. When the rescuer was looked
for she had disappeared. The man who was saved,
however, still hopes to meet his preserver and thank her
for his life, and facetiously expresses the desire that she
may be present on the occasion of his next funeral
procession with her restorative powers to win him back
to life."
AHuEgHi?8S,PiT900 "THE HOSPITAL? NURSING MIRROR. 265
CALLING IN THE NURSE TOO LATE.
In the second most satisfactory annual report of the
Wolverhampton District Branch of the Queen's Jubilee
Institute for Nurses attention is drawn to a point which
is not always sufficiently emphasised. The Ladies'
Committee remark that " it is often a matter of regret
that cases of serious illness, where good nursing is most
essential, are not made known until death is about to
take place, or, in some cases, has actually occurred."
The nurse, like the doctor, should be summoned as soon
as it is obvious that there is work for her to do. It is
to be feared that lives, which otherwise might be saved,
are lost from sheer inattention. This is the worst
result, but another unfortunate consequence is that in
such cases the nurse frequently shares with the doctor
the blame for an event which was inevitable at the time
she was called in.
THE SOCIETY OF TRAINED MASSEUSES.
The Council of the Society of Trained Masseuses
announce that their application for license of the Board
of Trade, which was advertised in the Times of June 2nd
and June 6th, has been accepted, and that the society is
now registered as " The Incorporated Society of Trained
Masseuses,'' Trained Nurses Club, 12, Buckingham
Street, W.C. The patrons of the society, and others
interested in massage, must feel gratified that the quiet
yet prosperous work of the society since its foundation
in 1894 has met with such success as to be now acknow-
ledged as a fully constituted body. The first general
meeting of the Incorporated Society was held on Friday,
July 27th, to which all the members were specially
summoned. The Chairman gave an account of the past
and present history of the society, and of the benefits
obtained from its being now an incorporated body; she
also read the list of officers and of those elected by the
founders to form the council; the financial position of
the society was also placed before the members, and the
names of certificate-holders who had already filled in
and returned nomination papers were enrolled as the
first members of the society. The society will work on
the same lines as before?holding examinations, grant-
ing certificates, establishing a registry, and in every
way trying to promote the efficiency and welfare of
masseuses?with the exception that all certificate-
holders will not in future necessarily be members of the
society unless nominated and elected by the council.
All certificate-holders, members and associates, will be
bound by the articles of association and the rules and
bye-laws of the society.
THE NURSES OF MILE END INFIRMARY.
The report of the examiner of the probationer nurse3
at the Mile End Infirmary is of a very pleasing charac-
ter, and was justly referred to the other day at a meet-
ing of the Mile End Board of Guardians by Dr. Brooks,
the medical officer, in terms of satisfaction. Quoting
the report, Dr. Brooks said that " the examiner was
agreeably surprised to find the standard of proficiency
so good, especially in the practical work. He felt that
whoever was responsible for both the practical and
theoretical teaching deserved credit for turning out
such proficient nurses. Each of the nurses qualified for
her certificate." This is not the only success scored by
the nurses. A charge of carelessness having been made
against a probationer, Mr. Loftus said he had heard
" frequent complaints from people wlio liad been in the
lunatic wards of bad treatment by the nurses." This
elicited from the Rev. Clark Hallam and other
guardians, an emphatic assurance that the nurses
treated the inmates of the infirmary, and especially the
lunatics, with the greatest kindness, and Mr. Loftus
found himself in a minority of one. As Mr. Hallam
observed, the evidence of lunatics would not be accepted
in a court of justice, and it is not fair to accept it
against nurses.
COOKING SCHOOL AT AN AMERICAN HOSPITAL.
Special attention is given at the State Hospital,
Binghamton, New York, to the art of preparing food
for the patients. The course of instruction is as
follows: One lecture is delivered each week to the
entire class, in which is discussed the composition of
food, its uses when taken into the body, the digesti-
bility of the different varieties and preparations, and
their adulterations, and the comparative values, both
nutrient principles and units of energy, of the varioua
foodstuffs on the market. The necessity for cooking
food is discussed at length, together with the contrasted
requirements of the two great divisions, proteids and,
carbohydrates, and numerous demonstrations are inter-
spersed, covering the four cardinal methods of cooking,,
boiling, roasting, broiling, and frying, and their different
combinations and variations. In the demonstration of
frying the different fats are discussed; their uses indi-
cated, and their burning point given, with practical
instruction in the methods of determining when the
various fats are at the proper temperature for frying to
produce the best results. During the demonstration of;
the methods of cooking various forms of starch the
composition of starch is discussed ; the effect of heat-
upon the starch grain, bursting.the sac which surrounds,
it, is explained, and the practical method of determining
when this has happened and the starch has been suffi-
ciently cooked for digestion is given.
THE QUESTION OF BEER MONEY.
The Wisbech Board of Guardians do not find it
easy to obtain nurses. At a meeting of the Board the
clerk stated that " all the nurses who had replied to
the advertisement of the Guardians had written to say
that they were either engaged or could not come."
Perhaps it was with a view to influence opinion favour-
ably that the Board subsequently adopted a proposal
to allow ?2 6s. per annum to the nurses who prefer to
go without beer. On a previous occasion a candidate
for an appointment who asked whether, being a total
abstainer, she would receive any allowance, was told
that " if she did not have beer she could not have the
money."
SHORT ITEMS.
Mr. Preston Thomas, Local Government Board
inspector, has reported Bodmin Workhouse nurse as
inefficient, but strongly supported the House Com-
mittee's recommendation that the Local Government
Board should be asked to sanction a pension of ?20
whereas if ten years?the Local Government Board
limit ? were added to her period of service she
would be entitled to only ?18. The Committee's
recommendation has been adopted.?The Portland
Hospital having finished its work, a number of mem-
bers of the hospital staff are coming home in the
" Canada," which is due at Southampton on Saturday.
Sister Cox Davies has been transferred, by request of
the army authorities, to work at Pretoria. Sister
Pretty, who had been seriously ill with enteric fever
and is reported convalescent, returns wit ten other
sisters in the " Canada."
266 ?' THE HOSPITAL" NURSING MIRROR. Sool
Xectures on IRursing for probationers.
By E. MacDowel Cosgrave, M.D., &c., Lecturer to the Dublin Metropolitan Technical School for Nurses.
XIII.?SECRETION AND EXCRETION.
The digestive canal and the different organs of the body
are lined with membrane made up of cells arranged some-
what as tiles are in a pavement. These cells are very
small, and can only be seen by the aid of a powerful
microscope ; but they have very important functions. In the
digestive canals it is these cells that absorb the food and pass
it on to the capillaries and lymphatics, but the cells have a
vital action; that is to say, they can not only absorb, but can
alter the food while it is passing through them. By this
vital action the cells are able to render the digested food fit
to circulate in the blood; this is necessary, as if fully-
digested peptones were injected into the blood without
passing through the cells they would act as a poison, and
even a small quantity would cause death. The cells can also
destroy any injurious matters which may be mixed with the
food, and thus often prevent germs of disease from reaching
the blood. The cells lining the digestive canal secrete mucus,
-and so the tissue composed of them is called mucous mem-
brane. But the cells can not only absorb matters, altering
nthem and passing them into the blood, but they can also take
"materials from the blood and form them into secretions. For
this purpose a number of cells are grouped together into a
gland.
A gland is formed by a tube of mucous membrane growing
down into the connective tissue ; sometimes, as in the gastric
glands of the stomach, the gland consists of the simple tube
of cells; sometimes, as in the sweat glands of the skin, the
tube is longer and its lower part is coiled up ; sometimes, as
?in the salivary glands, the lower part of the tube is
^branched, and the gland becomes of complicated structure.
In all cases the essential of a gland is a collection of cells
which have the power of manufacturing certain substances
from the blood. What comes from a gland is, properly
?speaking, a secretion, but commonly the word secretion is
,orly used when useful secretions are meant, the term
excretion being applied to secretions which throw off waste
matters. So secretions consist of substances which have
some useful action; these substances do not exist in the
blood, but are built up from the blood by the special cells
of the glands. They generally flow into the body. All the
digestive juices and milk are examples of secretions.
Excretions contain something injurious which existed in
the blood and needed to be got rid of ; the urea which is
excreted by the kidneys, and the carbonic acid gas excreted
by the lungs are examples. The cells of each gland can only
'form the secietion of that gland ; thus the cells of the breast-
gland always secrete milk, those of the liver always secrete
bile; so cells have a double power, they choose the materials
they require from the blood, and they alter those materials,
building them up into a new substance.
There are five important digestive secretions: (1) Saliva,
which flows from the three pairs of salivary glands?the
parotid, one in front of each ear; the submaxillary, under
the lower jaw ; and the sublingual, under the tongue. Saliva
digests starch into sugar by means of a ferment called
ptyalin. (2) Gastric juice, which flows from the tubular
gastric glands, which are so closely packed together that much
of the mucous membrane of the stomach seems almost
entirely made up of them. This secretion contains a little
hydrochloric acid and pepsin, by these it digests proteids;
it also contains rennin, by which it curdles milk. (3) Pan-
creatic juice, secreted by the pancreas; this contains trypsin
and other ferments, by which it is able to digest all classes
of foods. (4) Bile, which is secreted by the liver and stored
up in the gall-bladder. Bile assists the pancreatic juice to
emulsify fats, that is, to break them up into such small
atoms that they can be absorbed. (5) Intestinal juice, which
is formed by the glands of the intestine; it has but little
digestive power.
Excretions are necessary because the body is continually
undergoing change, its tissues burning with the oxygen
taken in by the lungs, and forming with the oxygen waste
products. Just as there must be an open chimney to carry
away the smoke, &c., from a fire, so there must be some
means by which the injurious products of combustion may be
removed from the body. This is done by the blood and the
excretory organs; the blood carries the injurious matters to
the excretory organs, which remove and get rid of them.
There are three sets of excretory organs : (1) The kidneys,
each being connected to the bladder by a long narrow tube>
the ureter. The function of the kidney is to secrete urine
from the blood ; this action goes on continuously, the urine
passing through the ureters into the bladder, where it
collects, being discharged
at intervals. In the kidney
the blood passes through
capillaries; these are in
contact with the kidney
cells, which pick up the
waste materials from the
blood. From two to three
pints of urine containing
an ounce of urea is excreted
daily. In diseases of the
kidney useful substances,
such as albumen, may es-
cape ; in other cases, the
kidney cells being diseased,
very little more than water
passes out, the poisonous
matters remaining in the
blood; these may cause
convulsions and death. The
more urine that is passed
the lower its specific gravity
is likely to be, as there is
the more water to dissolve
the solids. In hot weather,
and during exercise, much moisture escapes from the
skin and the urine often becomes so concentrated
that when it cools it deposits part of its solids.
The same thing happens in fever, the extra burning
requiring an extra amount of solids to be thrown off. (2) The
skin, contains many thousand sweat-glands, tubular in
shape, the part passing through the epidernrs being cork-
screw like, and the deeper part coiled up. The excretion of
these glands is called perspiration, and may be either insen-
sible or sensible. It is called insensible, when it passes
directly and invisibly into the air ; about a pint of vapour
escapes from the skin in this manner. If through exercise
or heat the flow is increased it appears as moisture on the
surface of the skin, and is then called sensible perspiration.
The sebaceous or fat glands also excrete ; they open into the
hair follicles. If the kidneys fail to act the skin can excrete
some urea, so in kidney disease it is often most important to
promote free action of the skin by wet packs, diaphoretics,
&c. (3) The lungs, which are the organs by which the
burned-up carbon of the body is excreted in the form of
carbonic acid gas. In twenty-four hours the weight of carbon
given out in this way is about eight ounces. Expired air also
contains moisture, about half a pint of water being thus
excreted daily.
Fig. 23.?Back View of Kid-
neys, Ureters, and Urinary
Bladder.
Aug.Tsflgoo! " THE HOSPITAL" NURSING MIRROR. 267
mursing on Boarb tbe " ?rcana."
THE STORY OF ONE OF THE SISTERS-IN-CHARGE.
By a CORRESrONDENT.
In the course of a chat with Sister McNeill, who was en-
thusiastic in her praise of the " Orcana," compared with
which, she said, " the other hospital ships looked like little
tubs when you see them together in the bay," I asked her
about the arrangements for the work of the nursing staff on
boxrd.
" We go on duty," she replied, " at eight o'clock, or earlier
if oar cases are bad. Breakfast is at nine, and we mess with
the officers. From two to five all but one sister go off duty,
and she takes orderly duty; it comes to each one's turn about
every fourth day. Then we go on duty again at five ; dinner
is at half-past six, and we go round again after dinner. At
nine we give in our reports both to the sister superintendent
and to the night sister. Sister Jeffreys took night duty. Her
hours were from nine to eight."
" And who is the sister-in-charge ? "
" Sister Mark, an Army Nursing Sister. The rest are
civilian nurses, five in number."
"You are assisted, of course, by the orderlies? How
many were there on board on your last voyage home?"
"About twenty-five. This time they were St. John
Ambulance men. In our first voyage home we had a number
of refugees as orderlies?civilians, all sorts of people who had
to leave the country, clerks, shopmen, and so forth. The
Work consists chiefly of fetching and carrying ; they helped
?with the food for the most part. The St. John Ambulance
men can help in the way of sponging the patients, &c., as
Well. I had two convalessent sergeants in my ward who
helped very much."
" How many doctors? " I asked.
"Three civilian doctors, under an R.A.M.C. Each doctor
takes charge of one ward, except in one case, where the
doctor had two wards under his care. They make their
rounds morning and evening, and more often if the cases are
' serious."
The "Nursery."
"What number of patients can the ship accommodate? "
" There are 180 cot3, and an officers' ward of six beds,
^"his time the officers were all very convalescent, and as they
Were all sub-lieutenants we called their ward the 'Nursery.' "
" And what are the wards like ? "
"They are beautifully arranged?beds in rows as in a
hospital. The cots can either swing or not, as the patient
Prefers. We have all the newest electric appliances; the
electric fans, for instance, are most useful for ventilating the
Wards. They are simply fixed when required, worked by
the electric current, and taken off again when finished with.
Electric kettles, too, are very convenient; we fix them on in
^e same way. The only thing to be careful about is always
have water in the kettle, else you burn a hole in the
bottom. The sterilisers also for the instruments are electric."
' Have you an operating theatre ? "
' Yes ; but we have not yet got the x rays. We had no
?perations on this voyage."
There are baths, of course ? "
" Yes, both salt and fresh."
And are the sisters' quarters nicely arranged ? "
' Yes ; they are most comfortable, and placed just between
he wards, in the middle of the ship."
"A Reae Pleasure Tiur, Sister."
. "Did any of the nurses suffer from sea sickness?" I
Squired.
"Oh ! yes, we nearly all did at the beginning of the
Voyage. But it makes no difference to your work, of course ;
you attend to your patients just the same, and then run
away ! Many of the orderlies were ill, too. I had the
misfortune to be really laid up myself part of the time, so
I know what it is like to be ill on board ship. The men,
however, enjoyed the voyage very much. 'It's a real pleasure
trip, Sister,' they said many times. And so it must have
been, after all they had gone through up country ? "
" Did they complain of their hardships 1"
" No; I never hear the Tommies complain."
"Such a Pretty Wound."
" I suppose that you had a great many convalescent fever
cases ?"
" Yes, and a great many rheumatism cases as well. Not
many were surgical. A few of the officers had gunshot wounds
in the feet, and one had had a bullet through the mouth ; it
entered at the right cheek, and came out through the jaw on
the left. But it left no injurious results. It was such a
pretty wound ! "
The Seamy Side.
" Did you lose any patients on the voyage ? "
"Yes, two of the men died. One was a sergeant; he
came on board with phthisis. The other was a lance-corporal
of the 2nd Middlesex Regiment; he died of enteric. He
ran a very high temperature for many days. His was
a very sad case, as he was quite young and had been
recommended for the Y.C. They both died the same night,
and were buried at the same time, one by a Roman Catholic
priest whom we had on board as he belonged to the Roman
Church."
" Did you go to the funerals? "
"No, I never go, it is too dreadful; so much more sad
even than an ordinary funeral on land I always think. Then
there was another most serious case of enteric whom the
P.M.O. did not think would recover; but he began to
improve slowly about ten days before we landed, and was by
that time in the convalescing stage."
How Tommy Amuses Himself.
" I suppose the men manage to amuse themselves very well
during the voyage ?"
" Oh, yes. Their concerts are very funny. They love
sentimental songs. There is one special favourite, about a
mother's grave?I wish I could remember the words; and
they are very clever with their hands, too. Nearly all of
them sew, and they make all sorts of things in wool. I have
a pair of baby's shoes which one of them made and gave me.
They had great fun at Las Palmas, where they bought
numbers of birds, chiefly canaries, and feathers to bring
home to their wives and sweethearts. Tommy is perfectly
happy if he has money to spend, especially as he is not
allowed to have charge of it on the voyage ; it is all given to
the P.M.O. to take care of. I am so sorry I did not see them
go off at Plymouth, for I pictured Tommy arriving with a bird
cage in one hand and his kit in the other. There was such
cheering when they went on shore. The sisters disembarked
at Southampton."
The Work.
" I conclude that you like army nursing ? "
" Oh, yes, very much indeed. I wanted, like many others,
to go to the front, and I came to Durban from Australia
?where I was trained?in the hope of being sent up country.
After three weeks' waiting, I got the offer of work on a
hospital ship, and accepted it. This is my second voyage to
England. We sail again on the 20th, and after that I should
very much like to go to China."
268 " THE HOSPITAL " NURSING MIRROR. Aug "wf'1900.
IRurstng in Hmcrtca: H IRejoinber.
By an American Graduate.
I should like, as an American graduate nurse, to erase
from the minds of English trained nurses the erroneous
opinion which "An English Nurse," in her short visit to
America, seems to have derived of our plan for obtaining
work after we have graduated.
The Register.
Every hospital to which there is a training school attached
for nurses also possesses a register or record book, where
each nurse as she successfully completes her three or four
years' training and obtains her diploma, enters her name.
Only those nurses who have graduated, and are in every
sense of the word " trained nurses," can have their names
entered here. As it approaches the date of final examinations
and graduations, the attending surgeons and physicians at
the hospital with whom the nurses have worked during their
term of training, having watched their progress, usually ask
them for their card, and give them permission to call on
them after they graduate. The register is kept either by the
superintendent or at the office. When an honorary physician
or surgeon attending that hospital or otherwise requires a
nurse, he can, by going there and seeing the register, make
his selection. As each nurse leaves her private case she
reports herself, when rested and ready for work, at her
hospital, and, unless specially asked for, her name is entered
and she again goes out in her turn.
The Training at St. Luke's Hospital.
Although Irish, I am very proud of my American
training, and very jealous for the hospital to which I
belong. I went to New York City, U.S.A., in the year
1887 to become a trained nurse. The American hospitals
were then taking the lead, and turning out for the nursing
world most thoroughly trained and competent nurses. I
entered St. Luke's Hospital, 54th Street and 5th Avenue, New
York City, and I feel I can never say enough in praise of
the thorough training given there, both in practical and
theoretical work. I have been kept more than fully occu-
pied by the doctors who knew me in St. Luke's ; I have never
had to apply even to my register for cases. I notice that
" An English Nurse " takes Boston as the centre of her few
weeks' knowledge; but from my 13 years' experience of
American nursing I cannot think the modus operandi of the
Boston nursing schools can be so vastly different to her sister
city New York. Our lady superintendent came to us from
the Massachusetts General Hospital in Boston; her views
did not then at all coincide with those of " An English
Nurse."
No Advertisements and No Nursing Homes.
We do not advertise. No. When we are thoroughly
trained our work is assured to us as we graduate by the
doctors who know us. Neither do we, for this reason, require
testimonials, unless we are taking up a field of labour else-
where. We have no nursing homes, I am thankful to say.
We make our own little homes, those of us who are alone in
America. And we are very happy, chumming two or three
together. When disengaged, we are glad to return to our
rooms and rest, tired out as we often are after a severe case,
nursed, most probably, single-handed. We are glad to go to
our own abode instead of returning to those comfortless houses
called " Nursing Institutions," or Homes, and of which I
have had some sorrowful experiences while in England. What
a very second-rate place "An English Nurse" must have
got into ! I have never heard of patients being charged a fee
to obtain a nurse, or vice versct. There are a few offices, I
know, where nurses minus a diploma, and so not recognised
by hospitals and others, go to try and obtain work ; but these
are not graduates, and I believe in such places a fee is
charged. Again, I am glad to say it has never been my expe-
rience to hear a graduate nurse called " a dreadful nuisance,
or considered an expense." My fees, 25 dols. to 30 dols.
a week, as the case might be, were always most willingly
paid, and my patients have ever been my truest friends.
Chumming Together.
When we graduate and take farewell of our happy hospital
life, two or three nurses usually chum together, take a flat
or rooms?where they establish themselves permanently?
get their cards printed, connect with the telephone, and when
we are quite sure we are suitably housed we then send each
doctor a card with the number of the telephone on it and nam?
and address, and the word "Engaged" or " Disengaged.1'
When going on or coming off a case we repeat this process for
the doctors' convenience.
American Quacks.
It would be interesting to know from " An English Nurse"
what kind of man she expected the American doctor to be.
If not a gentleman, her experience of medical practitioners
must, indeed, be a strange one ; it has never been my lot to
nurse for an American doctor who was not a gentleman, or
one who did not know how to respect a nurse. I am sorry
to say this has not been my experience while in England.
The English doctor seems to forget a trained nurse is, or
ought to be, a lady. He treats her in most cases with less
respect than he would show to his housemaid. Surely this
is not light. Again, I cannot fancy any medical man>
American or English, allowing a quack?as "an English
Nurse " terms the hypnotist?to interfere with any serious
case, pneumonia or otherwise, if the patient is under his care.
Neither is it possible to imagine a properly trained nurse
allowing a so-called "quack" to interfere. "An English
Nurse describes the experience as " horrible," yet she took
no means to stop the farce. Surely to any nurse a patient's
life is too valuable for her to risk it in such a way. Th0
nurse was in charge of the patient under the direction
her qualified medical man. She had his orders to ad-
minister brandy, but yet before she obeyed that order sbe
allowed her patient to grow rapidly worse, the farce
continue lialf-an-hour, and even then she deemed it neceS'
sary to obtain the quack's permission before she carried
out the orders of the doctor for whom she was nursing. ^
properly-trained American nurse would have allowed such 9
thing. In the absence of her doctor, her loyalty to both hi*11
and his patient, as well as her anxiety for her patient9
welfare, would have prevented such a humbug.
Specialisation.
How surprised "An English Nurse" seeni3 to be at tb0
American nurses being thoroughly trained. I must beg
contra diet her statement that everything is specialised. ^
is not so. We have wards for all diseases. My hospital ha
a splendid orthopaedic service, a children's ward of 60 bed?'
eye and ear service; maternity training was fully give0r
also fever, gynaecology, cancer, and specific diseases; ?u
surgical training was beyond reproach, and we ^velL
thoroughly taught to dispense our drugs and study tb?1^
effect. It is simply nonsense for " An English Nurso " toS
to the States for a few weeks and make such a statemeI1j
Our hospitals turn out magnificent nurses thoroughly
generally trained?very different from a number of Europ?7
hospitals, who charge a nurse fifty guineas, give her a 3*??/^
training, and send her out with a certificate a so-cal* ^
" trained " nurse, probably without ever seeing a casecfc,
enteric. I am proud to belong to that ideal hospital* ?
Luke's, and consider that the American nurses are in
respects very far in advance of their English sisters..
(To be continued
MTSSJ " THE HOSPITAL? NURSING MIRROR. 269
IRursing at tbc General Iboepital, Trieste.
A CHAT WITH THE AUTHORITIES.
By a Correspondent.
" I will give you a letter of introduction to the chief
doctor," said the British Consul, kindly, in answer to my
enquiry as to the best way I could go over the hospital and
hear about the nursing. Accordingly, in reply to the invita-
tion of Dr. X., the senior surgeon, I called the other morning
at the great building known as Ospital Civico. Upon my
arrival I was told that Dr. X. was operating in the theatre,
but another authority would see me. I was shown into the
Board room, and was most courteously received by Dr. A.,
the president of the Medical School.
" If you will ask me any question you like, I will en-
deavour to answer it," he said, and so I began :
"This seems a very large hospital; how many beds have
you ? "
"1,300," he replied. "To-day the number of in-patients
is 1,130."
'' And the number of nurses ? "
" Two hundred and fifty."
No Matron.
"Can I see the matron; it is chiefly about the nursing
that I am anxious to gain information ? "
" The matron ? Ah, I see you do not exactly understand
the system of our nursing, nor the status of the nurses here.
We have a head nurse, certainly, who superintends the
others, has charge of all the linen, &c.; but she is not the
kind of woman you in an English hospital would dignify by
the name of ' matron.' No; you had better let me answer
your questions, and afterwards I will send someone round the
hospital with you, and you shall speak to the ' matron' or
any nurse you choose. Then you will at once see what I
mean."
No Term of Probation.
"I suppose there is no regular system of training?" I
ventured.
" That is a difficult question to answer, because there is
and there is not! The fact is the nursing in this hospital is in
a state of transition. At present the nurses are drawn from
the lower classes. They are not bound in any way. Con-
sequently, some stay only a few months. After, say, six
months or a year, a nurse receives a testimonial. If she be
wise, and we approve of her, she stays on in the hospital.
In every ward the head nurse has been many years with us.
But we have as yet no regular term of probation. What we
should like would be to have educated women of a better
class, and to bind them for a definite period of training."
"I should have imagined that you could easily obtain
them in such an important hospital as this," I rejoined.
Tiie Language Difficulty.
"Mademoiselle, it is the language difficulty. In Vienna,
?r instance, they can have German nurses. Here, we must
have Triestines, who can speak Italian, and also the patois of
the people. But we hope, somehow, to be able to meet this
trouble, though it will not be easy in such a cosmopolitan
place as Trieste."
"Have you ever thought of giving the nursing into the
bands of a sisterhood ? "
A Sisterhood did not Answer.
"Until the year 18G0 the nurses here wero sisters. But
the arrangement did not answer, owing chiefly to the want
?f tact on the part of the sisters with regard to religious
questions."
Hours and Holidays.
" What hours do the nurses have? "
" There are generally six nurses to each ward of about
30 beds. We have three shifts in the 24 hours, so two are
on duty at a time. By this arrangement each nurse gets
several hours off duty daily; and each takes night duty
every third night. There is no regular staff of night
nurses."
" What about holidays ? "
" We have no settled holidays ; but the nurses can often
have half a day, a day, or several days, off duty."
Then the doctor spoke through the telephone to someone
in another'part of the building, and in a few minutes a man
appeared who was ready to take me round the wards.
We went through many men's, women's, and children's
wards. The " matron" I ran across in one of the large
corridors, and she kindly showed me much that was
interesting.
The nurses all looked strong, capable women. Their
uniform is odd ; it consists of a striped pink and white kind
of directoire coat opening over a grey cotton skirt; of caps,
collars, and cuffs they have none.
At this point I was obliged to leave my polite guides. I
should have been glad to have spent a good deal of time
looking at the wonderful kitchen and the laundry of the
hospital, to say nothing of the magnificent dispensary. Every-
thing is on the same gigantic scale. The civic hospital
possesses, too, every possible equipment in the way of clever
surgeons, who are keen scientists and rejoice in the latest
improvements. The only thing that seems out of proportion
is the nursing, and when the authorities are able to fulfil
their wish and obtain the services of a better class of women,
no doubt everything will be in harmony. That there is a
great future for the nursing department?once properly
arranged?of such a hospital seems certain.
H IDistt to Colesberg.
A member of the Army Nursing Service Reserve sends us an
account of a visit to Colesberg : " Two sisters and myself/*
she says, "asked and received permission for a day off duty
after five months' hard nursing, first medical and surgical,
lastly enterics and dysentery. We had been sending convoys
to Cape Town once and twice a week with convalescents, so
could be spared for one day. We were called by the night
sister at half-past four a.m. It was a fearfully cold morning,
ice on all the water. We had to catch the mail train, which
leaves the station any time between four a.m. to six a.m.
We steamed out about six a.m., passing on our way the
battlefield of Arundel and Rensberg, and a few other stations>
arriving at Colesberg Junction at half-past eight. Colesberg
is only 36 miles from our camp. We took a Cape cart to
Colesberg town, which is about two and a half miles from
the junction. The pretty little town reminded me very much
of Madeira. We breakfasted at the hotel, afterwards
making our way to the kopjes where the battles had been
fought. We reached a large expanse of veldt; on the left of
us were a range of kopjes which the Boers had occupied, and
on the right was Coleskop, the kopje where our men dragged
up two guns. One cannot imagine how they succeeded ; it.
is such a tremendous height. We picked up many bullets,
one shell, and pieces of shell, which we are treasuring. At
one p.m. we lound our way back to the hotel, and after
lunch drove to a kopje now named Suffolk Hill, where the
poor Suffolks came to grief. At the foot of the hill is a very
large grave where rest three officers and 27 men. Over the
hill, on the farther side, in a similar grave the Boers are
buried. I found several bullets on Suffolk Hill, but the hill
had been well searched, so that even bullets were few and
far between. From Suffolk Hill there is an excellent view
of the town and every kopje. We bought a few souvenirs
of Colesberg, and reached our camp again about half-past
ten p.m.
270 " THE HOSPITAL" NURSING MIRROR, Aug^s^im
3n flDemonant: J61i3abctb Grace Ibanan.
By One of Her Nubses (Miss Mary Gardner, Lady Superintendent of Birmingham and Midland Counties Sanatorium)
When Agnes Jones, first superintendent of nursing at the
Liverpool Workhouse Infirmary, lay dying, and her
physicians said that only a miracle could save her, a friend
rejoined, " Then a miracle will be wrought," convinced that
her life was so indispensable that the Lord of Life and Death
must restore her, even from the very gates of death. But
the lesson is continually taught that
" God doth not need
Either man's works or His own gifts."
Those who knew her personally, and the blank which her
loss occasioned, must have felt that her place could never be
filled; yet it was ere long nobly filled by her successor, the
subject of this memorial, who carried on the work begun by
her at Brownlow Hill Infirmary for some years, until invited
to undertake the superintendence of the new infirmary at
Crumpsall, near Manchester, then the largest hospital in the
kingdom.
TnE Scene of Miss Hanan's Life Work.
This was henceforth the scene of her life work. Here she
had to build up from the foundation, and the excellence of
the superstructure is testified to by the reputation of the
nursing school which she created. Her name is less generally
known than that of Agnes Jones, who, as the pioneer of
trained nursing in workhouse infirmaries, is entitled to the
chief place of honour, and whose early death, due to incessant
over-strain, cast a halo of martyrdom over her short career,
which was enhanced by the eloquent tribute of Florence
Nightingale to her memory. Miss Hanan's name and fame
are known to few beyond the sick among whom she worked
and the nurses whom she trained. But hundreds of the
latter, scattered far and wide, hold her memory in affection
and reverence, and count it their chief honour to have been
lier pupils.
Her Self-sacrifice.
Her life was also in some degree sacrificed. Possessing a
magnificent constitution she yet systematically over-worked
herself, until even her superb strength gave way, and
she retired after eighteen years at Crumpsall to be for the
short remainder of her life a suffering, helpless invalid. It
may be questioned how far such a course is right, but she
was truly a grea t woman in the exaltation of the ordinary
gifts and duties of womanhood.
The Head Nurse.
Her influence was a power in the hospital?felt by
all?by the nurses, who were for the most part her devoted
and loyal assistants, and by the sick, to whom she was ever
in truth as she loved to be called, the "head nurse." But
though respected by all and loved by many she could not be
described as "popular." The term is inappropriate. She
never sought popularity, yet she ruled the minds and hearts
of her subordinates as no merely popular leader could have
done. Miss Hanan's character was the most perfect combi-
nation of masculine strength with womanly tenderness it has
ever been my lot to meet. The greatest proof of the depths
of tenderness in that strong nature was shown by the fact
that many years of Poor Law service, with all its dis-
appointing, deadening influences, never hardened her. To
the aged and infirm and little children she showed exquisite
womanly tenderness. The most motherly natures are often
found in the ranks of the unmarried women. There is a
wealth of maternal feeling which never finds its natural out-
let, yet is not wasted, thank God ! In her case she became a
"motherof many."
A Personal Reminiscence.
How vividly I recall my first meeting with her when, a
shy, lonely probationer, sick with nervousness and apprehen-
sion, I lifted my eyes to the calm, strong face, whose likeness,
as seen then, is photographed on my memory for ever. My
first impression was of a magisterial presence, of one who
would be respected and feared rather than loved, but the
face, stern in repose, could light up, as I found, with the
loveliest smile, and the whole bearing soften. Her memory
is to me an inspiration, and her example what her teaching
ever was, " a counsel of perfection."
Her Ideal of Nursing.
She had the highest ideal of nursing, and her standard was
never relaxed. Therefore, she was often thought exacting or
"hard" by those who, oppressed by the sense of more work
than they could get through, felt that, like the Israelites,
they were expected to make bricks without straw, and mur-
mured accordingly. But it is just under such circumstances
that there is danger of slipshod performance, and a con-
viction that half-measures "will do," consequently the
greater need, especially in training, of a high standard rigidly
maintained. Many must have realised this later who had
found her test rigorous, and blessed the memory of their
teacher. She knew the difficulties in that great hospital,
where the nursing staff was for long so inadequate
to its needs. She worked strenuously for the increase of
the staff against inevitable opposition from the Guardians,
who as custodians of the rates are naturally adverse to
heavier expenditure, and her desire was accomplished. The
number of nurses has been increased by half since I knew the
hospital twelve years ago. Herself the hardest worker of
all, she had little time for personal intercourse with the
junior members of her staff, and this was a source of trouble
to her.
A Letter from Miss Hanan.
In a letter I received from her after her resignation she
wrote, " One of my anxieties and trials at the hospital was
that I felt I could do so little to help the young nurses. It
seemed to me with all I had to do I was unable almost to
speak to them just when in their first year's contact with so
much physical suffering and so many sad problems of life
they most needed helpful words, so that I am thankful when
anyone tells me I was able to help them in spite of
all. ... I begin to think much of the great meeting and the
full working time hereafter in God's purpose, where we
shall work without mistakes and without anxiety and
fatigue and pain." . . . Oh, brave noble heart, whose
teaching was manifested in daily life! Such lives as
hers are the strongest assurance to the doubting and
faint-hearted of the life beyond. She has left a legacy of
noble example and enthusiasm, which, working through the
live3 of others, will form her most fitting memorial, more
enduring than the cross of granite which it is proposed to
erect to her memory by the united offerings of nurses and
friends.
Her Life's Work.
She ever upheld the dignity of that branch of the nursing
service which she adopted and which owes so much to her.
It has been and is even now to some extent less popular than
general hospital nursing, for obvious reasons. But although
nursing in Poor Law infirmaries or State hospitals, as they
should rightly be called, includes much that is discouraging
and hopeless in its moral as well as its physical aspects, yet
the work has its brighter side, its rewards and consolations.
The matron of the Birmingham City Infirmary has well
said, "It appeals to those who care more for the human
than for the scientific side of their work." The work of
nursing as a whole was once classed in the lowest ranks of
unskilled labour. Florence Nightingale raised it to the rank
of a fine art. Following in her steps, Agnes Jones and
Elizabeth Grace Hanan led the reform in our State infirmaries.
Over the grave of the latter might be fitly inscribed,
" Life's work well done."
a" ""79%: "THE HOSPITAL" NURSING MIRROR. 271
HScboes from tbc ?utsibe Woilb.
AN OPEN LETTER TO A HOSPITAL NURSE.
A great sensation was caused by the announceme nt that a
plot to carry off Lord Roberts and murder his chief officers
had only been discovered and frustrated in the nick of time.
Even if the chief officers had not been murdered, the capture
of the Commander-in-Chief of our army in South Africa
would have been a most serious matter ; and it is very satis-
factory to hear from no les3 a person than " Bobs " himself
that the plot was "clumsily conceived." This must be
assumed to mean that Lord Roberts does not attach so
much importance to the incident as some of the London
papers. But all the same, one is glad to hear that the ring-
leaders and all concerned are under arrest. The question of
their punishment may safely be left to Lord Roberts, and
the persons who are demanding that they should be
hanged forthwith or shot, as the Times in a fine outburst of
frenzy urges, would do well to show a little self restraint.
The country which, with good reason, places implicit faith
in the generalship of Lord Roberts, will be slow to question
the wisdom of his administration because it is impugned by
a few ungenerous and half-informed critics at home. The
appointment of Field-Marshal Count Yon Waldersee to
the command of the Allied Forces, and the reported safety
of the Legations as lately as this week, are the chief items
of news in respect to China.
Like most great men, the Lord Chief Justice of England,
Who was buried on Tuesday, had his particular weakness.
He was passionately fond of gambling, and would bet on
anything, from a dinner to a race horse. But he did not
gamble for the sake of making money. It was purely a
pleasure to him, and up to his last illness he pursued it with
quite boyish ardour. Curiously enough, although his spirits
Were usually good, Lord Russell of Ivillowen was habitually
somewhat stern. His death was not expected by his family
until a few hours before it took place, and even Mr. Treves,
?who performed tho operation, with the view of saving life,
had hopes that his patient would survive.
Does the solidarity of hospital life ever impress you ? It
is sometimes exemplified in a very striking manner by readers
of this journal. For example, an article on a private hos-
pital in Bulawayo, in which some alleged defects were pointed
out by a correspondent, is, as you saw last week, construed
by the managers of the Memorial Hospital in that town as
an attack on their institution, and they hasten to defend it at
all points. A reference to the frequent resignation of members
the nursing staff at a hospital in Madras by a resident in that
?ity this week provokes a demand from home for a Government
inquiry. An account by an English nurse of her experiences
?f nursing in America, which was not unaccompanied by
criticisms, elicits an indignant rejoinder by an American
member of the profession, who proceeds to panegyrise the
hospitals in that country. That community of being which
hinds humanity into one whole, so that each affects and is
affected by all, is, in fact, particularly noticeable in hospital
life, and extends, apparently, to all parts of the civilised
World.
Having a few minutes to spare the other day, and finding
myself in the neighbourhood of the Marble Arch, I had
another look at the treasures in Manchester Square. It was
an exceedingly hot day, but Hertford House, with its lofty
rooms and big open windows, was refreshingly cool, and the
fountain playing in the courtyard behind also helped to sub-
due the heat. There were plenty of seats for the weary?
though backs are a luxury not allowed?and the quiet and
absence of crush, together with the wealth of works of art
upon which the eyes rested on every side, was distinctly
soothing after the turmoil of trains and omnibuses. Had I
been given my choice as to what I would have carried away
with me, I think it would have been a few of the antique
gems in some of the glass cases. They are so quaint in shape
and setting, the blending of the colours is so soft and artistic,
that I felt covetous in a way I should never feel
about modern jewellery, no matter how costly the gems.
Amongst the armour I noticed a Chinaman in full warlike
array, which especially interested me at the present juncture.
Not that the modern Chinese could fight in such splendid
garments. All his coat was studded with brass-headed
nails, and reached down to his toes, his stockings underneath
were of the closest needlework, and his tall, pointed helmet
of fine metal tracery, which was far too beautiful to be
"stormed at with shot and shell." I stood for a few minutes
and stared at the wax face of the Celestial, striving to-
imagine myself surrounded with hordes of the same type
thirsting for my blood, and the more I gazed the more thank-
ful I felt that I lived in England. Perhaps you may like to
know the hours for going to see Sir Richard Wallace's collec-
tion. Every weekday from ten to six free, except on
Tuesday and Friday, when there is sixpence to pay. Also
the exhibition is open until October on Sundays from two to
six.
Everyone knows that the present is a splendid time for
burglars to ply their unlawful trade, and the police are, of
course, specially on the qui vive to protect the empty houses,
but when trustworthy servants are left, and the robbing
process is carried out by means of sheer "cuteness" it is-
almost impossible for the authorities to be of much assistance
except to shut the door after the steed is gone. I have heard
of a good number of clever ruses, but this, which lately came
to my notice, struck me as particularly smart. A married
couple wished to leave town, and the lady offered her maid
board wages to go home for the time. The girl asked to
remain where she was, as her home was a long distance away,
and she said she had no fear of being left by herself. Nothing
unusual happened for a couple of weeks, and then one Satur-
day afternoon there was a ring at the front-door bell. The
maid, upon answering it, found a clergyman, who produced
a telegram, which ran as follows: " By all means use our
house. The servant is there and will make you comfortable.
Show her this," the signature being her master's. The " rev.
gentleman" explained that he was a great friend of the
family, was coming to preach in the neighbourhood, had
asked to be allowed to make use of the untenanted house,
and had received permission in the accompanying telegram.
The servant, although a little suspicious, felt that she could
hardly refuse to let the visitor in, more particularly as she
knew that her employer had a number of clerical friends.
She prepared a bedroom for him?where a little later
she found a cassock and surplice hung over a chair,
evidently removed from the bag?cooked dinner for him in
the evening, and then later on, having seen the guest enter
his bedroom, went to her own. She determined, however,
not to undress, but to listen. Soon after one o'clock the
quiet was disturbed by the gentle opening of the clergy-
man's door, and slipping out in her stocking feet she saw
him pause as if uncertain where to go next. He entered the
best bedroom, and she heard him quietly trying cupboards
and doors. Suddenly it llished across her that the key was
on the outside of the door. To pull it to and turn the key
in the lock was the work of a moment. The window was too
high from the ground for escape to be possible, and in a short
time, when the policeman came round on his beat, the thief
was secured and safely removed. He proved to be a
"gentleman " who had been long "wanted" by the autho-
rities.
272 "THE HOSPITAL" NURSING MIRROR. Ang.^mo!
IHovcIties for IFturses.
The Physiological Feeding of Infants. By Eric
Pritchard, M.A., M.D.Oxon: (London: The Scientific
Press. 1900. Price Is.)
Physiological Nursery Chart. Designed by Eric
Pritchard, M. A., M.D.Oxon. (London: The Scientific
Press. 1900. Price Is.)
Although of late years a vast amount of knowledge has
been gained in regard to the hand feeding of infanta much
?of it has, we fear, been but poorly assimilated even by medical
practitioners, while it is not too much to say that it has not
reiched the ordinary nursery at all. Nor can we altogether
wonder. To those who are within reach of such few milk
laboratories as (xist it is no doubt possible to obtain milk
modified according to the age and digestive capacity of the
baby, but in dealing with the ordinary nursery and the
ordinary mother or nurse any attempt to prescribe any modi-
fication of ordinary milk or to express in percentages the
proportions of the various constituents to be aimed at in the
mixture cannot lead to anything but muddle. Dr. Pritchard,
however, has very courageously tackled this complicated and
most important problem, and by working the actual amounts
of milk, cream, and sugar which are required to make a given
quantity (a pint) of food containing the various percentages of
the different constituents (fat, proteid, and sugar) which may
be wanted, he has reduced the whole affair to a series of recipes,
so that the practitioner has but to state what he wants and
the nurse, by the use of an ordinary ounce measure, can at
once produce what he asks for. The way the subject is dealt
with is admirable, and the intelligent use of the book and the
accompanying chart is likely to save many an infant's life,
and, what is perhap3 equally important, prevent many
diseases and disorders in after life which no doubt often have
their origin in the strain too often thrown upon digestion in
infancy. Many of Dr. Pritchard's remarks and observations
are of much interest and importance, and we especially note
his remarks about over-feeding and the risky condition
of very fat children. We have mentioned Dr. Pritchard's
book in this place because it naturally goes with
the "Physiological Nursery Chart," which has been
arranged by the same author, although we take it that much
of it is intended for the use of the medical practitioner. The
chart, however, is for the mother and the nurse, and if
regularly kept will serve admirably to show how baby is
getting on, and will undoubtedly in many cases give an early
warning when things are going wrong. Week by week for
the first year, and then month by month, spaces are pro-
vided in which to register the baby's weight, while full
directions are given for preparing the food required by the
normal child at various ages from the first day to the 18th
month. The intervals between the feedings, the total number
of the bottles, the quantity required for each feed, the quan-
tities in which the milk, the cream, the sugar, and the lime
water when required, are to be combined, are all given in
regard to each separate age, while many useful "nursery
aphorisms" are appended which the nurse will do well to
learn and ponder over. The whole thing is very complete,
and is sure to be both useful and interesting, for what can
be of greater interest when a baby is in question than to
watch its progress on the chart and note how it ig
' getting on ?"
PHYSIOLOGICAL NURSERY CHART.
(16 par <*?t. fa')
Ik eugar (by neuur*. %ct by
Total quantity for S4 boon (i
up by     ??*
p by additio* of waiar)
entity in each bottle (f
li lumber of bottlee ?
Intermit between feeding*
rS*""""' ?" ,i?"" te Jih"rf "" " NURSERY APHORISMS.
L Cox, 106 Hew Bond Street, Loodna, V
D?fe ot Birth,  ^  . 'weight chart.
b (be infill while foed-nq
m
^ e:::*
14. Dip the thermometer before the i
18. Mu; clotl.ee binder aoeeaeat and ca.
lOeelee.
19. Pure lir renders the blood pur*. Tel!
20. Let your nureery face lb* touth.
* tcalct; ordinary kitchen mica will antwer the purpote but the Standard Weighing Machine (XI It.), which nay be procured at A. Cot, 106 New Band Street, London, W . it
rf about < ox a. A fain of sore than 8 or baa thaa 4 on., if continued for xnl weeks in tueeeeetoo. may sea* that the child it being oeerfeJ. or requires nodifiootioa of the food. Cnder euch circumstance* the doctor thou Id bo oootulwd. ' Further
information at regards modi&catioo of food cay be found in " The Phytktiogical Feeding of lnfaata." Published by The Scmti&c Preaa, Ltd, print 1/?
?  />?**** *? g"* ***?*. M.A.. KD
ty 'C\* Sew*i^? Preee, Ltd., II eerf W Sin*, Jiraarf, i<mJ*
"THE HOSPITAL" NURSING MIRROR. 273
tlfoe IMurses' Boolisbelf.
[We invite Correspondence, Criticism, Enquiries, and Notes on Books
likely to interest Women and Nurses. Address, Editor, The Hospital
(Nurses' Book World), 28 & 29, Southampton Street, Strand, London,
The Magic Word. By Constance Smith. (Publishers :
Isbister and Co. 3s. 6d.)
The authoress of " The Magic Word " carries her readers
into far different scenes. Italy and South America are the
countries through which the story moves. An Italian prince,
awakened from a life of dilletantism to the realities of
existence by the sudden death of a young brother and the
desire to avenge it, finds also his salvation in the pursuit of
the man who is the cause of his death. This man is the
brother, unknown to him then, of the English girl he loves,
the girl who bravely stands between him and death on more
than one occasion, and finally, after many stirring and
perilous incidents, becomes his wife. There is no lack of
excitement in the twenty-three chapters which form the
book. The love scenes are touched lightly and delicately
enough, and a "Magic Word" will be very interesting to
those who know something of the unsettled public life of
South America, and the strong contrasts which life in that
country, socially and politically, present to the onlooker.
Carest Tiiou Not that We Perish ? The Bitter Cry of
Starving India. Illustrated by Original Photographs
taken in February, 1900, by Mr. and Mrs. Thomas A.
Bailey (late of Cork), Honorary Missionaries at Poona and
Bombay. By J. T. Budd, with Preface by the Rev. F.
B. Meyer, B.A. (London: Marshall Brothers, 10,
Paternoster How, E.C. Price 6d.)
This little book presents in very simple and unexaggerated
fashion some of the salient facts of the Indian famine. The
author gives full credit to the efforts made and the results
achieved by the Government in combating the distress, but
points out that there is also abundant room for private bene-
volence, especially in villages at some distance from the relief
camps, and for women and children. Not only food but
clothing is wanted for these; but the author wisely points
out that European cast-off clothing is wholly useless for
Indian wearers. Instructions are given for the making of
the ? sari " of the Hindu women, and the " chudder " and
other garments worn by Mohammedans. But gifts of
Material for clothing is more desirable than garments made
here, as caste prejudices, strong even in the last extreme of
necessity, make some unwilling to wear clothes made by
Europeans, or some difference in style from the familiar
pattern may make a garment useless to one of a nation bound,
like the Hindus, by the cast-iron chains of immemorial tradi-
tion. Best of all are donations of money for the purchase of
home-made material, is local manufacturers are suffering.
Mr. Budd also dwells on the importance of spending wisely
the money gathered for the relief of the famine-stricken, re-
commending especially the development of irrigation works,
which tend to prevent future famines, and are, even as a
commercial speculation, often very profitable. The photo-
graphs with which the booklet is illustrated show very
clearly the deplorable condition to which many of our Indian
fellow-subjects are reduced by famine.
Life in a Provincial Workhouse Hospital. By Annie
S. Pruett. (Thomas 0. Todd, Sans Street, Sunder-
land. Pp. 42.)
This little work, without pretending to the possession of
any literary ability, is nevertheless an interesting record of
daily routine life in a workhouse infirmary situated in a busy
Provincial borough. From it we obtain an insight into the
class of cases to which such institutions minister, and about
which the public at large have but a very hazy idea. Life in
a country workhouse must always be more or less monotonous
from a nurse's point of view ; there are no accidents of the
nature met with in our large general hospitals to be attended
to, and no visiting surgeon making his daily round with a
band of students to whom he lectures at the patient's bed-
side. It must not, however, be assumed, as Miss Pruett
points out, that therefore the cases do not require equal care-
and attention. It is in a nurse's skilful management of suchu
patients that her abilities most conspicuously shine forth, re>-
quiring, as she does, ceaseless devotion and self-denial, coupled
with no little firmness, endurance, and high principle. In the
introductory chapter a wholesome warning is given against;
young women taking up such work unless actuated by the
highest motives. The following chapters give a sketch of the
daily work in the wards, which is pretty much th&
same everywhere, and only differs from established use
in the details. In the provincial workhouse a good dea)
more responsibility in the way of first aid and diagnosis of
cases would appear to be vested in the superintendent than
is usual in the larger infirmaries where there is a resident
staff of medical officers. For instance, on page 38, in the
case of a" fit" she must be able to recognisa what kind of
fit it is, and render the necessary assistance till the doctor
comes. Should an accident occur in the workhouse the nurse
is sent for, and is empowered to use her judgment in removing
the patient to the infirmary. In Chapter IX. we read that
" the nurse is called frequently to the different parts of the
workhouse to an inmate taken suddenly ill, or to an inmate
who has met with an accident," and till the doctor arrives is
authorised to take such measures for the relief of the suf-
ferer as she may deem to be expedient. She also is liable
to be called upon to visit the tramp ward to decide whether
its occupant is a malingerer or not, an invidious distinction,
and one which if she is wise she will leave altogether in the
hands of the medical officer. It is, on the whole, a readable
little book, and we have pleasure in recommending it to the
notice of all those who are interested in the way in which the
State discharges its duty towards the sick and infirm in our
workhouses and workhouse infirmaries.
?be Ibanan Memorial jfunfc.
Major Ballaxtine, master of Manchester Workhouse.
Crumpsall, asks us to publish the following second list of
subscribers to theHanan Memorial Fund : Miss Turner, 103.;
J. W., Is. ; Nurse Clarke, 2s. 6d.; Nurse Tomlinson, 2s. 6d.;
Nurse Tomlinson (second subscription), 2s. 6d.; Nurse
Kernaghan, 5s.; Nurse Campion, 10i.; Nurse Kidd, 5s.;
Nurse Carlisle, 53. ; Mrs. Bostron, Sweden (by Nurse
Piggott), ?2; Nurse Milliken, 2s. 6d. ; Mrs. Dobson, 10s. 6d.;
Nurse Chevallier, 2s. 6d.; Nurses Mary and Grace Morton,
10s. Gd. ; Nurse Scholes, 5s. ; Nurse Fazackerly, ?1; Nurse
Boddington, 10s. ; Nurse Whitehead, 53. ; Mrs. McWilliam,
5s.; Nurse Warriner, 10s. 6d.; Nurse Macfarlane, ?1 ; Nurse
Currie, 5s.; Nurse Macmanaway, South Africa, ?1; Nurse
Head, 5s.; Nurse Gardner, 5s.; Mrs. Sattin, 5s.; Nurse Blox-
ham, 53. ; Nurse Ruttledge, 5s.; Nurse Hunt, 5s. ; Nurse
Colfin, 10s.; Nurse Hudgson, 5s.?total, ?12 153. The
subscription list will be kept open up to the end of this
month.
appointments.
Cook's Terrace Infirmary, Pancras Road, London.?
Miss Elizabeth Jane Blake has been appointed Nurse-
Matron. She was trained at St. Marylebone Infirmary, and
has since been night supeiintendent at St. Marylebone
Infirmary and home sister at Crumpsall Infirmary,
Manchester.
New Joint Hospital, Spring Yale.?Miss Jessie Mao-
gillivray has been appointed Matron. She was trained at
Kilmarnock Infirmary for three years. She has since been
sister in charge of fever wards, and subsequently matron of
Thomas Walker's Hospital, Fraserlaugh, Aberdeen.
Paignton Cottage Hospital.?Miss Evelyn Hilton has
been appointed Matron. She was trained at Newark and
Charing-cross Hospitals, and has since been matron at the
Brighton, Hove, and Sussex Throat and Ear Hospital.
274 " THE HOSPITAL ? NURSING MIRROR. Aug
jpiaoue^lftursing in tbe ITlp^Countr^
By a Nurse Correspondent.
I was one of two who had asked to go up country, to a
station whence a call had come for two sisters.
So one Monday afternoon a few days later we started from
Bombay by the two o'clock train, going by way of Poona.
Just outside that town we ran into a thunderstorm, and in
Poona station we dined to the accompaniment of heavy rain,
which came down with a sort of unrelenting vehemence
one never hears at home. For the coming night's journey
we got a little sleeping carriage to ourselves, and there we
slumbered peacefully in our berths, only awaking next
morning to find that we had journeyed through the night
into a land of spring. Below and before us lay stretched a
country of young up-springing grain, and ever and again trees
in freshest green, and over all the dew and pureness of a
new morning. However, in spite of tbe enjoyment of things
outside, we were not altogether sorry when we drew up at
our breakfast station, and were able to get out and stretch
our legs and see someone beside ourselves. The guard wires
on orders for meals from a station some way from the one
where the breakfast, tiffin, or dinner is to be. When you
are new to the country the appearance of the guard at your
carriage window, asking you if you want breakfast or dinner,
rather takes you by surprise. That morning we were a party
of seven for haziri.
Our Quarters.
At about six the same evening we arrived at our destination,
where we found the collector awaiting our arrival, and he and
others most hospitably entertained us. They lent us their
servants and sent us chota haziri, that is, your first or " little
breakfast," which you usually have in your room ; and they
took us to breakfast and dinner, until finally we got settled into
our quarters and then began work. We had " tossed up " for
choice of duties, the result of which was that I went on at night.
The hours we arranged from seven to seven. Accordingly,
one evening I drove down to the hospital just before that
hour. At that time we had a night staff of three ward boys
and two ayahs, none of them, of course, trained. There is
also a hospital assistant living in the camp, a native who has
had two years' training in a civil hospital and three years' at
one of the recognised medical schools out here. The quarters
of " the lady nurse in charge," as we are officially called,
consist of two rooms in one of the two principal bungalows,
and there, that first night, I was half-an-hour or
more writing up reports. On going over to the big bunga-
low, imagine the horror of an English-trained nurse at
finding all her nursing staff asleep, scattered all over the
verandah?one a bundle of white under the table, some on
the ground, others on chairs, and yet another on the dispensary
table.
Our Patients.
We had seventy-five patients to begin with. The cases
were really decreasing as we came ; there had been as many as
one hundred and eighty, and the wards had been over-
crowded. The patients are put into huts, or wards in the
bungalows or tents, but the latter, as far as possible, are kept
for convalescents. The big bungalow facing the entrance
gates contains five wards, besides the dispensary and the
steward's office?the two latter being at either end of
the verandah. Like all bungalows, it consists of a
large central room, opening into and surrounded by
smaller ones. The central ward has eleven cots, and
the smaller make up fourteen more between them.
The cots are rather curious specimens of furniture, a frame-
work of four poles, which fit into four more upright ones?
the legs. Across is a web of cord, the bed bars and mattress
in one; the whole?the native bed or "charpoy." When a
patient comes in, two covers or blankets are fetched from the
cupboard, grey and red ; the former is laid on the cot, while
the latter covers the pitient. Besides the bungalows, of
which there are three altogether, we have two more wards,
partitioned off by matting into huts, which contain one or
more cots. At the present moment in one of these are two
sepoys, a policeman, and a forest guardsman ; the former
is very aggrieved that he has not got a hut to himself. But
I rather fancy that this is a grievance that will not be
remedied.
The Convalescents.
The convalescents live in the tents, which are somewhat
apart from the wards, and the night nurse in charge knows
little of them. They are for the most part happy people
who slumber through the night hours, without rude awaken-
ings for temperatures and milk, or " cludh," as they call it.
A visit to the tents, or indeed to any part of the camp,
requires the attendance of a boy with a lamp or " butti,"
in order that one may avoid stepping on snakes ; but I have
never seen anything worse than a toad hop out of the darkness
occasionally.
These warm tropical nights are not without their charm,
especially when the moon is up. It gives a clearer and
stronger light than at home; clear enough at the full to read
your glass by. But often the night is so busy that there
is not time to enjoy the charm, and before one is aware it is
dawn?a dawn so short that it seems to end almost as soon
as it has begun?and the sun is with us again and all things
are wide awake on the instant. Shortly after Sister " Mem-
sahib " (for they give us brevet-rank in the wards) goes to
bungalow for breakfast and bed.
(Ibe IRurstno of paupers.
WHAT THE GOVERNMENT INSPECTORS SAY.
A fortnight ago we published some interesting extracts
from the report of Mr. Baldwyn Fleming, the inspector for
the district comprising the union counties of Dorset and
Southampton and part of Wilts and Surrey. We regret that
several of the inspectors under the Local Government Board
make no reference at all to the nursing question in their
reports, while others do not deal with it in anything like an
adequate manner. If the subject had been treated generally,
as Mr. Fleming has treated it, the result would have been an
illuminating mass of evidence of the greatest value.
Salop, Chester, and Parts of Hereford, Stafford,
Worcester, and Derby.
Mr. Iv. L. Dansey, inspector for this district, reports that:
"The Nursing Order of 6th August, 1897, has had a marked
effect for good throughout the district, and, although I dare
not say that the order has not been occasionally infringed by
pauper attendants trespassing upon the nurses' ground, yet
the boundary line is now getting more recognised, and the
medical officers are more prompt to act under section 5 of the
Order in requiring skilled help when necessity arises. The
obstacle to such help is generally want of accommodation for
nurses, and this is too slowly being provided. There is still
much to be done to secure a proper staff of permanent nurses
at most workhouses, for, while in some cases there is one
nurse to 12 or 1G beds, in others there is only one nurse to
3G or 38 beds, and the average for the whole district is only
about one nurse to 21 beds. But it must not be assumed
that the number of beds correctly represents the 'number of
patients, for in many workhouses, especially the smaller
ones, probably not moro than half the beds would be occupied
by day and night, although in the larger urban workhouse
hospitals the proportion occupied would be considerably
more."
Lancaster and Westmoreland and Parts of Cumberland
and the West Riding of Yorkshire.
Mr. H. Jenner-Fust, inspector for this district, says:
"The 'Nursing Return,' which is now annually issued to
Ang.^900.' "THE HOSPITAL" NURSING MIRROR, 275
?every guardian in this district, shows a steady increase in
^he number of nurses as compared with tho number of
patients. In five years the total number of nurses employed
*n the district, including superintendent nurses, assistant
superintendents, charge nurses, assistant nurses, and pro-
bationers, has increased by 199, and the number of patients
per nurse has fallen from 1 in 17 to 1 in 13, the return for
January 1st, 1899, showing 9,08-1 patients under the care of
the medical officers (exclusive of imbeciles and epileptics in
special wards), attended by G64 nurses. The number of
attendants on imbeciles and epileptics placed in special wards
also shows an increase, there being on January 1st, 1899, one
attendant to every 20 patients, as compared with one to 20
patients five years previously."
The East Riding of Yorkshire and Parts of the West
Riding.
Mr. H.G. Kennedy, inspector forthisdistrict, mentionsthat
at Keighley a small detached nurses' home has been finished
and brought into occupation ; that at Sculcoats the new nurses'
quarters are in occupation; and that at Sheffield a new
nurses' home has been provided. On the general question he
says : "Important additions to the nursing staff were made
various workhouses during the year under review, and
duly qualified superintendent nurses?conformably to the
provisions of the recent Nursing Order?are now on duty at
several infirmaries where no such officers existed before."
Cornwall and Devon and Part of Somerset.
Mr. Preston-Thomas, inspector for this district, only
makes a general allusion. He says that "the old
folks in the infirmaries are well fed and housed, are
Carefully nursed, and have medical attendance when
necessary, have only such light work as serves to occupy
^hem, and enjoy ample liberty as to visiting and being
Visited. If they belong to a distant parish it often happens
fchat a relation or friend is brought on market day in the
cart of some kindly farmer to see them, for most of the work-
houses are placed in market towns. In the infirmary itself,
too, they often find enjoyment in each other's society; and
"?vhere the master and matron are thoughtful and j udicious
^here is often some attempt at classification which has the
effect of bringing together persons of like character, and of
Preventing the respectable from being annoyed by the dis-
putable."
North and South Wales and Monmouthshire.
Mr. F. T. Bircham, inspector for this district, writes : " It
is satisfactory to be able to report that the connection
between guardians and district nursing associations, in North
^Vales especially, is steadily being developed. With the
amount of outdoor relief that is given in this district, there is
almost unlimited scope for this much wanted work. As it is
n?t the practice of the various boards?with the exception
a few of the Urban unions?to send their sick into
Workhouse infirmaries, so they should at least justify their
action by rendering it possible, as far as is in their power,
fchatthe outdoor sick should receive proper skilled attention
^here required."
Co IRurses.
, E invite contributions from any of our readers, and shall
J* glad to pay for " Notes on News from the Nursing
World,? or for articles describing nursing experiences, or
paling with any nursing question from an original point of
lew. The minimum payment for contributions is 5s., but
welcome interesting contributions of a column, or a
p.ge, in length. It may be added that notices of enter-
^ainments, presentations, and deaths are not paid for, but,
course, we are always glad to receive them. All rejected
^manuscripts are returned in due course, and all payments for
JUanuscripts used are made as early as possible at the
eginning of each quarter.
ffov IRea&iitfl to tbe SlcU.
"I will lift up my eyes unto the hills, from whence cometh
iny help."?Ps. cxxi. 1.
When sick of life and all the world?
How sick of all desire but Thee !
I lift mine eyes up to the hills,
Eyes of my heart that see?
I see beyond all death and ills
Refreshing green for heart and eyes,
The golden streets and gateways pearled,
The trees of Paradise.
" There is a time for all things," saith
The Word of Truth?Thyself the Word?
And many things Thou reasonest of;
A time for hope deferred.
But time is now for grief and fears ;
A time for life, but now! is death;
Oh, when shall be the time of love,
When Thou shalt wipe our tears ?
Then the new heavens and earth shall be
Where righteousness shall dwell indeed ;
There shall be no more blight nor need,
Nor barrier of the sea ;
No sun nor moon alternative,
For God shall be the light thereof;
No sorrow more, no death, no sting,
For God who reigns is love. ?C. Bossetti.
Reading.
When I look into this blue sky it seems so deep, so
peaceful, so full of mysterious tenderness, that I could lie for
centuries and wait for the dawning of the face of God out of
the awful loving-kindness.?G. Macdonald.
Into our lives in many simple, familiar, homely ways, God
infuses this element of joy from the surprises of life, which
unexpectedly brighten our days and fill our eyes with light.
He drops thi3 added sweetness into our cup and makes it to
run over. The success we were not counting on, the blessing
we were not trying after, the strain of music in the midst of
drudgery, the beautiful morning picture or sunset glory, the
unsought word of encouragement or expression of sympathy,
the sentence that meant for us more than the writer or
teacher thought?these and a hundred others that everyone's
experience can supply are instances of what I mean. You
may call it chance or accident?it often is ; you may calf it
human goodness?it often is; but always, always call it
God's love, for that is always in it. These are the over-
flowing riches of His grace; these are His free gifts.?
S. Longfellow.
He will never fail me. What He now is to me, that He
always will be. The Lord stands around His people "from
this time forth for evermore." Sooner will the hills remove
from about Jerusalem than the Lord will remove from about
His people. Jerusalem saw many troubles, but there stood
the hills still. I too have seen troubles, I am in trouble
now, and I may yet see even greater troubles ; but the Lord
will not remove from me. The hills could not always protect
Jerusalem, but the Lord will always protect me.
Lord, give me the eye of faith, to see Thee always near
" I will lift up mine eyes unto the hills, from whence cometh
my help. My help cometh from the Lord, which made
heaven and earth."?Bourdillo
There's a land where suffering dies for ever,
" A quiet habitation," where our Lord
Will lead His chosen by a still, calm river
And pastures fair?so saith the Eternal Word.
There will the sick the great Physician meet,
And healed rest for ever at His feet.
? C. M. Prevost.
276 "THE HOSPITAL" NURSING MIRROR.
Cver?bo&?'s ?pinion.
[Correspondence on all subjects is invited, but we cannot in any way be
responsible for the opinions expressed by our correspondents. No
communication can be entertained if tlie name and address of the
correspondent is not given, as a guarantee of good faith but not
necessarily for publication, or unless one side of the paper cnly is
written on.]
BEWARE OF "THE CARRIERS' ACT."
" Nurse G." writes : In removing last month from
Folkestone I handed to the agents of Pickford and Co. in
that town some luggage, among which was a strong, well-
locked and strapped metal trunk for conveyance to Syden-
ham. On my arrival I found that my trunk had been picked
and my money, watch, and valuables stolen; also that an
inner writing-case had been broken open. After three weeks
have elapsed I am coolly told by Messrs. Pickford and Co.
that they are by " The Carriers' Act protected from liability."
This, I fear, is true, but it teems a flagrant injustice that
any Act of Parliament should enable the thief, whether in
the carriers' employment, or in that of the railway company, to
commit larceny with impunity. I can only hope that my
severe loss may induce my fellow-nurses never to place
money or other valuables in their trunks.
OUGHT NURSES TO DO SO MUCH MENIAL WOEK ?
"J. R." writes: Some will say that no work which a
nurse does is menial and that nothing ought to be beneath
the dignity of a nurse, but one cannot help noticing the
amount of work a nurse has to do in hospital which really
has nothing to do with nursing. We know that cleanliness
is very essential, but still ought not the wardmaids (who
very often get less menial work than the nurses) to have the
actual cleaning part to do, such as sweeping, scrubbing
lockers, &c., and let the nurses have a better opportunity of
seeing the real side of nursing ? I know it may be said that
it is quite as necessary for a nurse to know how to clean,
otherwise how is she to superintend others. Still it seems
rather hard that a nurse must, in order to become thoroughly
trained, also go through a course of real charwoman's woik.
Does this really help very much towards making her a good
nurse? Does it not rather help to keep down the standard
of nurses instead of raising it ? A big part of the remedy
lies in the hands of those who superintend.
IS UNIFORM TO BE COMPULSORY?
Nurse Beatrice writes : The sentiments expressed by
"Pavo" in "The Nursing Mirror" re nurses' uniform
will, I am sure, meet with a responsive echo in many a
heart. Of course, one great advantage in wearing uniform
lies in the fact of its being quickly donned, and this
alone has great weight with such busy women as we must
needs be. But even so I do not see why we should be com-
pelled to blazon our profession so continually before the eyes
of the world, more especially as so much has been brought
before our notice lately with regard to the abuse of our dress,
which ought to be sacred to a fully qualified nurse only.
What should we say to a civilian donning a soldier's uniform ?
And what are nurses but soldiers, fighting against the great
enemies of mankind, viz., disease and daath? I am afraid
much cannot be said about the unobtrusiveness of uniform,
as we constantly hear it being spoken of as " so attractive."
In fact a great many girls are accused, unjustly I hope, of
entering the profession for no higher motive than the
ambition of being able to wear it. Of course, we are all
aware that the dress is merely a survival of the time when
all nurses were nuns. I think it behoves us all to be most
careful to keep our dress as neat and quiet as possible, and
not to forget that we hold the honour of a noble profession
in our hands.
THE GENERAL HOSPITAL, MADRAS.?A GOVERN-
MENT INQUIRY ASKED FOR.
" A Member of the Committee or a Large Provincial
Hospital" writes to us as follows: I am very glad to see a refer-
ence to the above hospital in " The Nursing Mirror " of July
28th, for it is high time attention was drawn to the unsatis-
factory state of things which exists, and which is most
inimical to the well-being of both the nursing staff and the
patients. "A Resident in Madras" evidently writes with a
knowledge of the facts in stating that the real difficulty with
regard to the nurses is the " keeping them." If the official
records of the General Hospital were examined it would be
found that during the last two or three years a quit?
abnormal percentage of the English nurses and probationers
have resigned prematurely, although by so doing they have
everything to lose professionally. Some nurses leave
without giving any reason, while others perhaps state
" work too hard," or some excuse of that kind \
but privately they all assign quite other reasons for
leaving, and generally express astonishment that any
self respecting English lady can consent to remain at
" The General" under the conditions which prevail.
Each of these nurses could, like " A Resident in Madras," a
tale unfold ; and a tale which in the best interests of an im-
portant hospital (500 beds) ought to be unfolded. Unfor-
tunately moral courage appears to be at a low ebb under the
enervating climate of Madras; so, rather than face an un-
comfortable quart d'heure or have to state unpleasant truths,
nurses and others leave without putting on record their real
reasons for doing so. Those reasons are well-known to a
number of people in Madras, both inside and outside the
hospital, and are, I believe, not unknown to some past anu
present members of the Madras Council. Unfortunately, the
"General," being a Government hospital, appears to lack
that essential safeguard of good management, a local com-
mittee ; the result is that nurses can only make their
grievances known to Government through certain official
channels, with the strong probability that such complaints
will be strangled by red-tape in passing through such a
medium. I venture to suggest that Government should
institute a searching inquiry by an impartial tribunal
(entirely independent of the hospital staff), to ascertain if the
treatment and training of the nursing staff are what they
ought to be, and the general management and tone of the
hospital satisfactory. Will Sir Henry Burdett place this
important institution and the nursing community under a
still further obligation to himself by taking up this question -
fBMnor appointments.
Cook's Terrace Infirmary, Pancras Road, London.-^
Miss Maud Alexander Foster and Miss Lydia Harris Foster
have been appointed Sisters. Miss M. A. Foster was trained
at St. George's Infirmary, Fulham Road, and has since beeO
ward nurse at Camberwell Infirmary and head nurse at
Woolwich Infirmary. Miss L. H. Foster was trained at
Hackney Union Infirmary. She has since been probationer,
assistant, and staff nurse in the same institution, and sister
at Woolwich Infirmary. Miss Matilda Beer, Miss Ada
Annie Stallard, and Miss Lilian Agnes A. Challender have
been appointed Staff Nurses. They were all trained at St*
Pancras Infirmary. Miss Stallard has been nurse at the
Foundling Hospital.
Tcnbridge Wells General Hospital.?Miss
Swingler has been appointed Night Sister. She was traine
at the Children's Hospital, Great Ormond Street, London,
and South Hants Infirmary, Southampton. Miss Mario0
Robertson has been appointed Children's Ward Sister. Sb0
was trained at the Royal Hospital for Children, Edinburgh*
and Charing Cros3 Hospital.
Maidenhead Workhouse.?Miss Minnie Davies has been
appointed Assistant Nurse. She was trained by the Mea
Workhouse Nursing Association at St. Peter's Home*
Mortimer Road, Kilburn.
Aug."goo!' "THE HOSPITAL? NURSING MIRROR. 277
IRotes ant? ?ueries.
The Editor is always willing to answer in this oolnmn, without any
fee, all reasonable questions, as soon as possible.
But the following roles must be carefully observed :?
1. Every communication must be acoompanied by the name and
address of the writer.
2. The question must always bear upon nursing, directly or in-
directly.
If an answer is required by letter a fee of half-a-crown must be
enolosed with the note containing the inquiry.
South Africa.
(192) Will you let me know, as soon as convenient, if a nurse with in-
feotious training, not general, could at present get a post in South
Africa ??A. C.
It is improbable that a nurse without general training .would be ad-
mitted to the Army Nursing Service Reserve.
Further Training.
(198) For four years I have been engaged in infectious nursing, and
am desirous of receiving 12 months general training. Age 25. Do you
think I may obtain a post as senior probationer in hospital or infirmary,
or would my previous experience disqualify me ??F. S. E.
You ought to be able to find what you want without difficulty, as you
are fully trained in one branch of nursing. The real difficulty is as to
Setting a conjoint certificate.
Ear Hospitals.
(194) Will you please let me know the address of one or more of the ear
hospitals in London, and by what means a non-paying patient can get
admission to same as an out-patient ?? Nurse if.
The Central London Throat and Ear Hospital, Gray's Inn Road ;
admission free. The London Throat Hospital for Diseases of the Throat,
Nose, and Ear, 204, Great Portland Street, W.; admission free or by
Payment.
Short Sight.
. (195) Will you kindly tell me if wearing glasses will prevent my obtain-
'"g a position as probationer in a hospital ? I wear glasses for short
sight, otherwise my eyes are strong.?Else L. G.
Short sight is not always a bar to a candidate's acceptance. The
question can only be definitely answered at the medical officer's
examination.
Two Tears' Training.
(196) Please tell me the address of any hospital or infirmary that will
take a nurse for two years, who already holds a certificate for one year,
where a certificate would be given ??E. M. B.
The Cancer Hospital, Fulham Road, Brompton; the Bootle Borough
Hospital; the Kent and Canterbury Hospital, Canterbury; the Derby
j^nion Infirmary; the Newcastle Royal Infirmary; and the Sheffield
*wyal Hospital, and Royal Infirmary, Sheffield.
Tonbridge Worlchouse Infirmary.
(197) Will you kindly inform me if three years' training given at the
tonbridge Union Workhouse Infirmary is recognised by the Local
government Board. I see on one of their printed forms that midwifery
training will be given and nurses prepared for the examination of the
?London Obstetrical Society as opportunities arise ??Sissic,
Write to the Secretary of the Local] Government Board, Whitehall,
S.W.
PrivateJNursing.
(198) I am a fully-trained nurse and would like to do private nursing
f?r the winter in Egypt (Cairo), or would go into hospital there. Will
y?i kindly tell me to whom I should apply ??Anxious.
Apply to the Matron of the Deaconess Victoria Hospital, or the Head
Sister at the Kasr-el-Aini Hospital, Cairo, either of whom might be able
help you.
Open-Air Treatment for the Poor.
(199) At what places in England is open-air treatment of consumption
Carried out for the poor ??Matron.
At the Hospitals for Consumption at Hampstead and Yentnor, and
aleo at the National Sanatorium, Bournemouth. Write for particulars.
Trained Nurse.
j, (200) I am anxious to become a trained nurse. I am 19 years of age.
j^you^thmk I could get into a hospital P I am strong and healthy.?
The only institutions which will take you at 19 are the children's
hospitals.
Queen's Nurses at Brighton.
(201) Could you kindly give me the address of the Queen's nurses at
Brighton ??H. J. II.
A letter addressed to the care of the Superintendent of Queen's Nurses,
t. Katharine's Precincts, Gloucester Gate, Regent's Park, N.W., will be
forwarded.
Training in Scottish Poor-Law\Infirmaries.
infl ^ have recently entered as a probationer in a large poorhouse
jnnrrtiary in Scotland, and I should like to know before going further if
, v P00rhouse infirmaries in Scotland are fully-rocognised training
an- ^ I gain my three years' certificate here am I eligible for an
, ppomtment in any of the general hospitals in the United Kingdom; or,
?lng trained in a poorhouse infirmary, am I only eligible for appoint-
ments in such ??U. B. II.
All general hospitals have their own separate and independent rules,
^t as to whether any individual poorhouse infirmary is recognised as a
fnai2ill? school, inquiry should bo made at the Local Government Board
Scotland, and also for England, both Boards having hitherto declined
0 18sue any general list of the training institutions which they recognise
Matron in an Orphan Asylum.
(20S) Will you kindly tell me if general training is necessary in order
to obtain a post as matron in an orphan asylum ? Also, what other
qualifications are necessary, and if a well-trained nurse could obtain
suoh a post without influenoe ??Nurse.
Different regulations are made by different committees. General
training would be of use anywhere, and, as a rule, there is no difficulty
in obtaining such a post without influence. Good health and recom-
mendations are, of course, essential.
A Question of Procedure.
(204) Will you kindly tell me whether a doctor can refuse to attend a
case when called in by a nurse ??Maria.
A doctor can do exactly as he likes, whoever calls him in.
Army Nurse.
(205) I should very much like to be an army nurse. Will you kindly
tell me if I must be a probationer in a general hospital before I could
enter a military one P?F. E. N.
Tou cannot become an army nurse till you have had three years' train-
ing in a general hospital and obtained your certificate.
The Sot-Air Treatment.
(206) Will you kindly tell me if the hot-air treatment can be carried out
in a private house without the patient having the journey and expense of
going to London, &c. ? Would the cost be very great, and is it possible
to gain instructions in the treatment in a short time ??A Superintendent.
Certainly it'ean be done in a private house. The question is altogether
one of expense only. Application should be made to Mr. Tallerman,
9, Welbeck Street; The Dowsing Radiant Heat Company, 24, Budge
Row, E.G.; and Mr. Greville, New Bond Street, W.
Red Cross Nurse?Beading Up Cases.
(207) 1. What are the essential qualifications for a Red Cross nurse ?
2. I should be glad if you could advise me as to a suitable book contain-
ing most cases in a large general hospital at a reasonable price.?
Inquirer.
1. A nurse who works under the Red Cross Society must have had tbe
necessary training. During the war in South Africa general civilian
nurses have been employed by it. 2. Nursing cannot be learnt from
books. But useful books to read are : " A Hand Book for Nurses," by
Dr. Watson (Scientific Press); " Nursing, Its Theory and Practice,"
by Dr. Lewis (Scientific Press); and "A Manual of Nursing, Medical
and Surgical," by Laurence Humphry.
Lady Resident Medical Officers.
(208) I am anxious to enter a good general hospital as probationer.
Will you kindly inform me if, as it has been suggested to me, the ap-
pointment of lady resident medical officers will diminish the value of the
certificate of an institution ??Irish Nurse.
We do not think that the fact to which you refer makes any difference.
The Salary of a Masseuse.
(209) Can you tell me what is a fair salary for a masseuse in a large
establishment ??Massage.
Apply to the Society of Trained Masseuses, 12, Buckingham Street,
Strand, W.C.
" Laryngitomy."
(210) 1. Is there such an operation as larotomy ; if so, what is it done
for ? 2. What is laparotomy usually done for??A Constant Reader.
1. If you refer to laryngitomy, that means cutting into the larynx?a
sort of tracheotomy. 2. Laparotomy is performed when a surgeon
requires to open the abdomen.
Projecting Ears,
(211) I am very much interested in a pretty girl whose beauty is com-
pletely spoiled by projecting ears. Can anything be done to remedy
this ? Would it be possible to have them stitched back ? She was a
seven-months baby, so perhaps this accounts for it. In the case of her
ever marrying, do you think her children, if she had any, would inherit
this disfigurement ??Inquirer.
Consult a specialist on deformities.
Tow.
(212) Will you kindly tell district nurse the best means of making tow
straight from the mill antiseptic ??Jean S. S.
Cleanse thoroughly by washing in soda and water and keep in a car-
bolic lotion until required. But to render it aseptic the simplest plan is
to heat it in the steriliser.
Probationer.
(218) I should like to know if there is any hospital which I can enter
as probationer at the age of 19 years, and receive a salary ? B.
Only the children's hospitals will receive probationers under 28, and
none under 20. The commencing salary at 20 is from ?10 to ?12.
Holiday Home.
(214) Can you inform me if there is a holiday home for nurses at
Scarborough ??I. M. ........ a . , ...
No doubt the matron of the hospital at Scarborough would give you
the information you desire. There is a convalescent home and home of
rest for ladies. The address is St. Martin's Lodge, South Cliff. The
payment is 15e. per week.
Standard Books of Reference.
" The Nursing Profession: How and Where to Train." 2s. net; post
free, 2s. 4d.
?? The Nurses' Diotionary of Medical Terms." 2s.
" Burdett's Series of Nursing Text-Books." Is. each.
" A Handbook for Nurses." (Illustrated.) 5s.
" Nursing: Its Theory and Practice." New Edition. Ss. 6d.
" Helps in Sickness and to Health." Fifteenth Thousand. 5s.
All these are published by The Scientific Pkess, Ltd., and may be
obtained through any bookseller or direct from the publishers, 28 i 29
Southampton Street, London, W.C.
278 " THE HOSPITAL" NURSING MIRROR. AugMMMO.'
travel IRotes.
LV.?ROUEN.
In writing these papers my great object is to give them the
greatest variety, and so to cater for all tastes?for those
whose purses are slender and play-time limited, for others
to whom time and money are no object, for the seekers after
health and the comfortably robust, for the artist and the
Philistine, for those who prefer beautiful scenery to any-
thing else, for those whose chief delight is in wandering
amongst the beautiful architecture of the past, and for those
who think the chief delight of a holiday is to tear wildly
about 60 miles a day on a bicycle or 20 on their feet, and last,
but not least, for the saunterers who like to take their
holiday without undue exertion. Therefore, if sometimes the
theme seems to an individual reader uninteresting, I must
beg for indulgence. A mighty cyclist will think it a poor
thing to potter round Rouen, but pity the weaker vessels
and let them have their chance.
Expenses of the Journey and Accommodation.
Via Newhaven and Dieppe your fare will be first-class
return ?2 5s. 3d., second ?1 13s. 6d. You may live very
comfortably for 6 frs. a day at the Hotel de la Couronne or
at the Grand Hotel du Nord I think, but always jwrite first
and make an arrangement. For a prolonged stay it is quite
likely that you may agree upon even less if you do not mind
a small room high up. It would be well to stop an hour or
two in Dieppe to see the beautiful church of St. Jacques,
with its quaint surrounding market and the old castle. That
is all of antiquity that is left. Dieppe has become very
modern.
The Sights of Rouen.
These are only such as will please the lover of the antique
and picturesque?the artist, the photographer, and the
archaeologist. The entire charm of Rouen lies in its mediaeval
architecture, which carries us so far back in thought to the
days cf long ago. Much of its interest in this respect has
departed; the terribly utilitarian spirit of the nineteenth
century has not left Rouen untouched; it makes one tear
one's hair and gnash one's teeth to think what artistic trea-
sures in the way of domestic architecture have been ruthlessly
swept away for the birth of the Rue de la Republique and its
tram line. However, if you turn up the Rue Eau de Robec
you will still be charmed with the tumbledown gables and
windows at all angles that delight the lover of the picturesque.
The Cathedral and St. Ouen.
The cathedral of Rouen is one of;the most glorious speci-
mens of its kind to be found in France. I like to advance
towards the south portal as the light begins to fade. Inside
and outside are alike exquisite. There is a most beautiful
effect of columns and shafts in the nave, which Murray
thus describes: " Suspended between the base of the
columns which support this gallery and the capitals of the
aisle piers are exquisitely beautiful clusters of five detached
and bended shafts projecting on a ledge." The windows,
especially the rose in the west front, are beautiful in form,
though some of the glass is mediocre. I like particularly the
wheel window in the north transept. There is in the choir
the tomb of Richard Coeur de Lion, but as to the present
whereabouts of his heart opinions differ. The sacristan
avers it is below the pavement safe and sound, but the
gardien of the Musee, nothing daunted, shows you a casket
with the rescued relic enshrined. The two most beautiful
tombs are those of Cardinal D'Amboise and Louis de Breze.
There is a an ornate Gothic stone stair leading to the library,
which is indeed a thing of beauty. Many people
consider St. Ouen superior to the cathedral, and
in some ways it is so, but its position is far
from being so striking. It is larger than the cathedral,
and very graceful in its proportions, but to my mind not so
absolutely satisfying to contemplate. The west door will
not bear comparison with that of the [cathedral, but the
south portal is worthy of minute attention ; it is a gem of
sculpture, and is called Des Marmosets, on account of the
animal life depicted on the gargoyles. The wheel window in
the north transept has a tragic history. Alexander Berneval,
haying entrusted the execution of this window to a pupil,
killed him in a fit of envy and jealousy because the
work surpassed his own. He was executed for this crime,
but the monks allowed him to be buried in the church (in
St. Agnes' Chapel) because he had contributed so much to its
beauty. Look into the holy water stoup near to the west
door; it reflects in a very striking manner the whole interior
of the building. It is sometimes covered by a board, but it
is permitted to move it.
St. Maclon.
This church is a favourite of mine, principally because it
has such delightful cloisters and quaint buildings attached
to the presbytery. Into these hallowed precincts?the
courtyard of the priest's house, indeed?I unwittingly
entered, and was violently ejected by a lady armed with a
huge ladle. She was entirely deaf to my humble apologies,
and I was put to the rout, horse and foot. The great feature
of St. Maclon is the door on the north side, carved by Jean
Goujon ; the subjects did not seem to me very terrible, but
the conscientious sacristan hurried us on lest the young
lady's mind should be contaminated. This he explained to
me behind his hand in a windy whisper.
Hotel de Ville.
Follow the main street east from St. Maclon, and you will
come to the Hotel de Ville ; there is nothing to be seen
inside. Continue the street to the Fontaine Ste. Marie, well
worth seeing.
The Grosse Horloge.
This is one of the quaintest things in Rouen, and no doubt
you are well acquainted with it in pictures. All round it
are to be seen some of the best existing specimens of domestic
architecture?the Tour St. Andre, the Yieux Marche, and
especially
The Hotel Bourgtheroude,
which is close to the Place de la Pucelle, where Joan of Arc
was burned. The Bourgtheroude is in good preservation, and
has a wonderful frieze running round the courtyard repre-
senting the meeting of Henry VIII. and Francis I. on the
Field of the Cloth of Gold. You will make acquaintance
with much that I cannot now tell you of. Do not forget that
there are three beautiful fountains to be seen, all Gothic?L?
Croix de Pierre, La Fontaine de la Crosse, and La Fontaine
de Lisieux.
TRAVEL NOTES AND QUERIES.
Belgium for French (Aspirant).?I should not myself clioose
Belgium, tlie French is naturally not so good as in its own country-
Certainly do not go to Bruges, there are far too many English; some
very quiet out of the way place like Courtrai, Alost, or Ypres, would b?
better. I do not think you would find some quiet French central town0
beyond your means. Such as Ohartres or Bourges. Tours has a nam0
for very pure French, and there are not so many English there now as &
few years since; still it is much more expensive than Bourges or Ohartres-
Further Eouth is cheaper still, but in the Midi the French is not so pure-
There are many small places where you can live very cheaply, but tor
acquiring perfection in a language, you need to enter into a fcomewhftt
fuller life than you can do in a village.
Madrid, taking Burgos en route (Gyp).?Yes, Burgos is on tu?
mail route, and can be seen easily in one day. Travelling in Spain 1?
slow. Leaving Paris at 10.30 p.m., you will reach Burgos at 8.44 tne
next night, or Madrid at 7 the following morning. There is not very
much to be seen at Burgos except the cathedral, but that is superb.

				

## Figures and Tables

**Fig. 23. f1:**
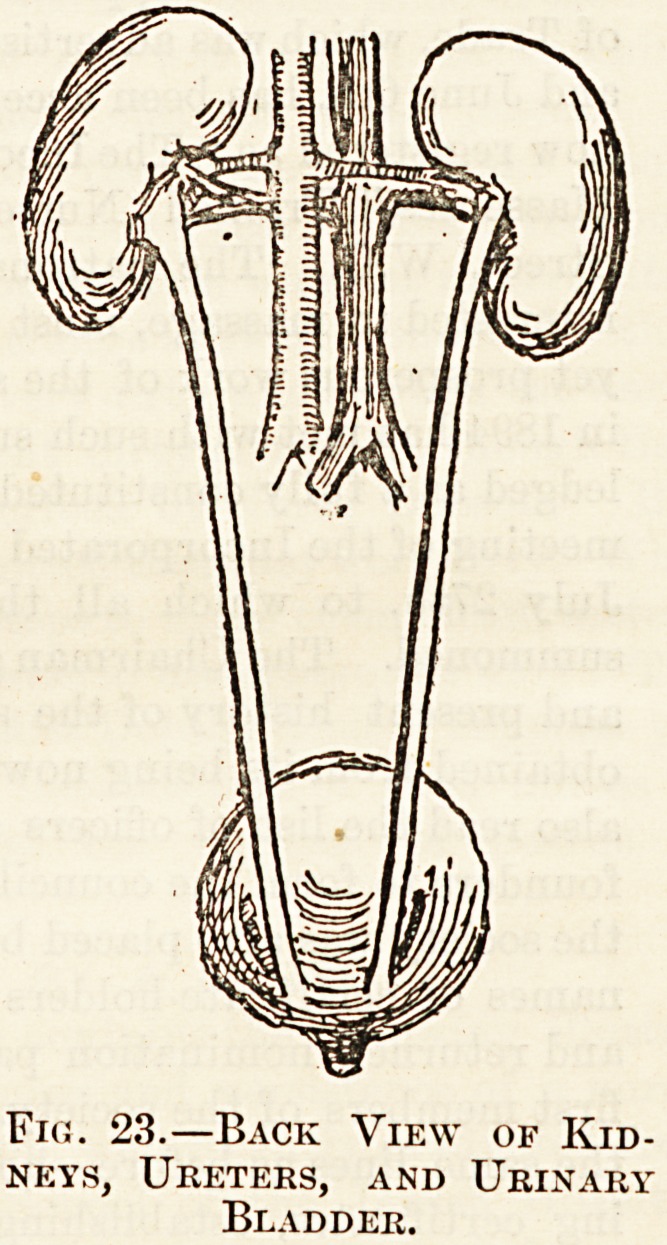


**Figure f2:**